# The quality changes in fresh frozen plasma of the blood donors at high altitude

**DOI:** 10.1371/journal.pone.0176390

**Published:** 2017-04-21

**Authors:** Zongkui Wang, Hua Liu, Miaomiao Dou, Xi Du, Jijun Hu, Na Su, Ya Wang, Rong Zhang, Changqing Li

**Affiliations:** 1 Institute of Blood Transfusion, Chinese Academy of Medical Science, Chengdu, China; 2 Tibet Autonomous Region Blood Center, Lhasa, Tibet; 3 Guizhou Taibang Biological Products Co., Ltd, Guiyang, Guizhou; Food and Drug Administration, UNITED STATES

## Abstract

**Objective:**

According to the international guidelines, fresh frozen plasma (FFP) is unanimously used to treat coagulation disorders. The quality of FFP is critical for the clinical transfusion. Till now, few studies have integratedly evaluated the differences of FFP from blood donors at between high altitude (HA) and low altitude (LA). Besides, there were no special quality standards for HA FFP in China.

**Materials and methods:**

Up to 41 HA (Lhasa, 3700 m) and 46 LA (Chengdu, 500 m) blood donors were included in our study to estimate the differences of FFP from HA and LA blood donors. The concentration of total plasma proteins, prothrombin time (PT), activated partial thromboplastin time (aPTT), thrombin time (TT), fibrinogen (Fbg), factor (F) II, FV, FVII, FVIII, FIX, FX, FXI, FXII, D-dimer, protein C (PC), protein S (PS), antithrombin III (ATIII) and von Willebrand factor antigen (vWF:Ag) were determined, respectively.

**Results:**

As compared with FFP of LA blood donors, the total protein content of HA blood donors showed a significant decrease (65.2±8.9 vs.57.2±6.3 g/L; p*<*0.001); PT, aPTT, TT were significantly increased (p*<*0.001); the levels of FII, FV, FVII, FVIII, FIX, FX, FXI, FXII and vWF:Ag were notably decreased (all p*<*0.05), whereas Fbg and D-dimer were dramaticly increased (p = 0.038). Additionly, in HA blood donors, vWF: Ag and FVIII:C of O-group was significantly lower (p<0.05) than that of non-O-group. It should be noted that FVIII:C of HA blood donors (0.64±0.10 IU/mL) was lower than the current Chinese quality requirements for FFP (≥ 0.7 IU/ml). No significant differences were observed in PC, PS and ATIII.

**Conclusion:**

In general, our findings showed that the quality of FFP was significantly different between HA and LA blood donors, and the current Chinese quality requirements of FFP are not suitable for HA FFP. Therefore, setting up a special quality requirement for HA is quite necessary and meaningful.

## Introduction

Low ambient temperature and hypobaric hypoxia are two challenges to life at high altitude (HA) [1]. Extended stay at HA may be a risk factor for development of symptomatic portal system thrombosis [2]. As compared with low altitude (LA) residents, people who stay at HA were found with prolonged clotting time, decreased FVIII activities, increased protein C (PC) levels and significantly changed D-Dimer, von Willebrand factor (vWF) activity [3–5]. All of these reports indicate that HA may influence the quality of blood products.

Fresh Frozen Plasma (FFP), which contains normal levels of stable clotting factors, protease inhibitors, immunoglobulins and albumin, is always used for hereditary and acquired coagulation disorders, thrombotic thrombocytopenic purpura, disseminated intravascular coagulation, warfarin reversal, massive transfusion, and so on [6–9]. The quality of FFP directly affects the efficacy of clinical transfusion. In China, there is no special quality standard for HA FFP. Despite the fact that there is quality requirements for FFP in China (the activity of factor (F) VIII is at least 0.70 IU/mL and the content of total plasma protein is at least 50 g/L [10]), however, no one knows whether the quality requirement is suitable for HA areas

In order to verify whether the current FFP quality requirement is suitable for HA blood donors, the present study was conducted to investigate the differences of the levels of total plasma proteins and clotting factors in FFP of blood donors at between 3,700 m (Lhasa, HA) and 500 m (Chengdu, LA). Furthermore, vWF antigen (vWF: Ag) and FVIII:C in HA blood donors of different genders and blood types were also analyzed. Based on the observed results, additional data could be provided for transfusion services at HA and help clinicians to use FFP more rationally and effectively.

## Materials and methods

### Ethics statement

The study was approved by the Ethics Committee of the Institute of Blood Transfusion. All procedures concerning the experiments with human plasma had been given prior approval by the Department of Public Health of Sichuan Province. All participants gave verbal informed consent, and the copy of the informed consent was left with the participant.

### Study design and sample preparation

Forty-one HA and forty-six LA residents were enrolled in this study. HA blood donors were recruited from Lhasa, Tibet (3,700 m) who reported no visit to LA areas and LA blood donors were enrolled from Chengdu, Sichuan (500 m) who reported no visits to HA in the six months prior to this study. Inclusion criterias were that all volunteers were ≥ 18 years, healthy and unrelated. Individuals who had prior history of thrombus or hemorrhage, usage of oral anticoagulation therapy, hepatic disease, HIV infection, pregnancy, diabetes, renal insufficiency and others were excluded from this study by standardized questionnaire according to the “whole blood and component donor selection requirements” [11]. The basic information of blood donors were shown in Table 1.

**Table 1:** Demographics of blood donors in this study.*

	n	Age	Gender		Blood group			
			male	female	A	B	AB	O
HA donors	41	32.3±9.7	23	18	5	13	6	17
LA donors	46	35.9±8.0	22	24	14	16	7	9

* Data are presented as mean ± SD.

According to the Chinese quality requirements for whole blood and blood components [10], blood samples were collected by venipuncture. The whole blood, within 6 hours after donation, was centrifuged at 4,000 x g for 20 min to obtain the plasma. Then, the fresh plasma was stored at -70°C until they were shipped to the laboratory at Chengdu in dry ice type environment. Upon arrival to the laboratory facility, the samples were immediately stored at -70°C until analysis. All samples were measured in laboratory at Chengdu. Samples showing evidences of hemolysis and/or clot formation were discarded.

### Laboratory analysis

Hematological parameters (i.e., red blood cell (RBC) counts and platelet counts) were assessed by using an automated hematology analyser (Sysmex XE 2100, Kobe, Japan). Total plasma protein content was determined by the method of Bradford using coomassie blue G250 as the staining agent. PC (Hyphen BioMed, Neuville-sur-oise, France) and antithrombin III (AT III) (Sekisui Diagnostics, LLC, Stamford, USA) were measured with chromogenic substrate assays, and VWF:Ag was performed by using an enzyme-linked immunosobent assay (Hyphen BioMed, Neuville-sur-oise, France) with SpectraMax M2^e^ (Molecular Devices, Sunnyvale, CA, USA). All other assays were performed according to manufacturer’s instructions on a CA-1500 automated coagulation analyzer (Sysmex Corporation, Kobe, Japan). FII, FV, FVII, FVIII, FIX, FX, FXI, FXII, D-dimer and protein S (PS) reagents were purchased from Siemens Healthcare Diagnostics Products GmbH (Marburg, Germany). Prothrombin time (PT), activated partial thromboplastin time (aPTT), thrombin time (TT) and fibrinogen (Fbg) reagents were from Chengdu Union Biotechnology Co., Ltd. (Chengdu, China).

### Statistical analysis

Kolmogorov–Smirnov test was used for the normal distribution of all data, and values were expressed as means and standard deviation (SD). Results of HA blood donors were compared to that of LA blood donors by means of independent sample Student’s *t*-test. A robust analysis of total plasma protein and FVIII was given by receiver operating characteristic (ROC) curve. Statistical significance was defined as *p*<0.05 and the software used for the statistical analyses was SPSS statistics software, version 17.0 (SPSS Inc., Chicago, USA).

## Results

### Counts of red blood cells and platelets

RBC counts showed a significant increase in HA participants, as compared with the values measured in LA volunteers (Table 2). However, platelet counts were lower (p = 0.017) in HA than LA blood donors (Table 2).

**Table 2:** Counts of red blood cells and platelets.^§^

Analytes	LA	HA	*p*-value*
RBC (10^12^/L)	4.61(10IN	5.26(10IN	< 0.0001
PLT^#^ (10^9^/L)	198.98018.55	181.31011.23	0.017

^**§**^All data are presented as mean ± SD;

^#^PLT, platelets;

*LA blood donors compared with HA blood donors using independent sample student’s *t*-test.

### Total plasma protein content and basic coagulation tests

The results of total plasma protein content and basic coagulation tests (PT, aPTT, and TT) were displayed in Table 3. Compared with LA blood donors, the total protein content of HA blood donors was significantly decreased (*p<*0.001), whereas PT, aPTT,TT were significantly increased (*p<*0.001).

**Table 3:** The total plasma protein content and basic coagulation tests results of different altitude blood donors.^§^

Analytes	Reference range	LA	HA	*p*-value*
total plasma protein (g/L)	≥50	65.2±8.9	57.2±6.3	*<*0.001
PT (sec)	10–15	12.6±1.5	14.7±1.4	*<*0.001
APTT (sec)	22–38	30.4±4.2	39.4±5.9	*<*0.001
TT (sec)	16–18	16.0±1.4	17.9±1.5	*<*0.001

^§^All data are presented as mean ± SD.

*LA blood donors compared with HA blood donors using independent sample student’s *t*-test.

### Coagulation factors of different altitude blood donors

**Fig 1:**
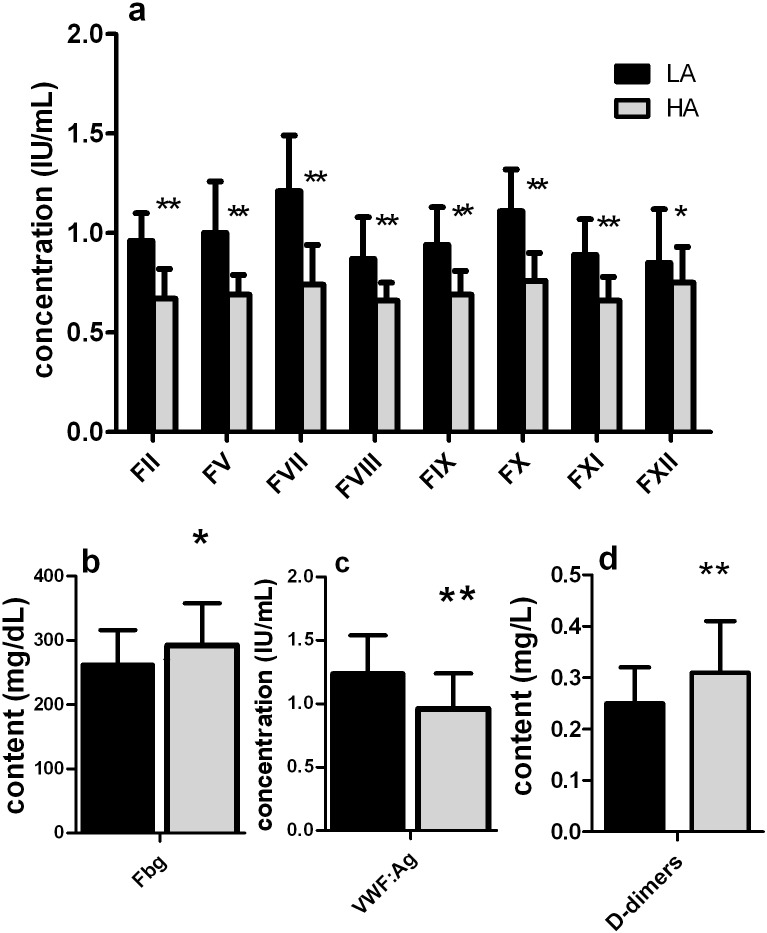
The results coagulation factors of different altitude blood donors. LA, blood donors reside at low altitude (500 m); HA, blood donors reside at high altitude (3,700 m). Values are mean ± SD. LA, n = 46; HA, n = 41. **p<0.001, *p<0.05, when compared LA blood donors and HA blood donors using independent sample Student’s *t*-tests.

The results of coagulation factors (i.e. FII, FV, FVII, FVIII, FIX, FX, FXI, FXII, Fbg, D-dimer and vWF: Ag) were summarized in Fig 1. As represented in Fig 1A and 1C, vWF:Ag and all coagulation factors were significantly higher in LA than in HA blood donors (*p<*0.05), whereas Fbg and D-dimers significantly increased in HA blood donors (*p<*0.05; Fig 1B and 1D). FVIII:C of LA blood donors was significantly higher than that of HA donors (0.87±0.21 *vs*. 0.64±0.10 g/L; *p<*0.001).

### Anticoagulant factors of different altitude blood donors

Table 4 displayed results of anticoagulant factors (i.e. PC, PS and ATIII) of different altitude blood donors. PC, PS and ATIII showed no significant changes (*p>*0.05 for all comparisons).

**Table 4:** Anticoagulant factors of different altitude blood donors^§^.

Analytes	Reference range	LA	HA	*p*-value*
PC (%)	70–140	106.1±24.1	100.7±20.6	0.244
PS (%)	60–130	88.1±21.2	81.8±24.5	0.382
ATIII (IU/mL)	0.8–1.2	1.01±0.26	0.98±0.17	0.215

^§^All data are presented as mean ± SD.

*LA blood donors compared to HA blood donors using independent sample student’s *t*-test.

### VWF: Ag and FVIII:C of HA blood donors

**Fig 2:**
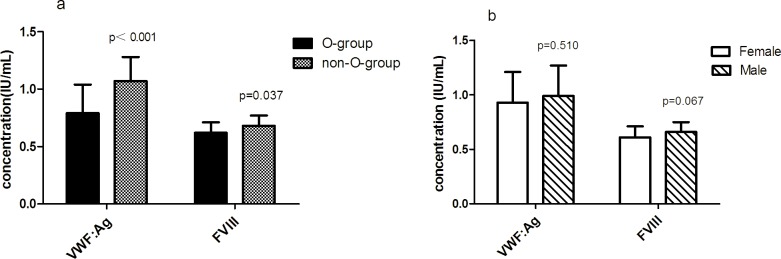
Levels of vWF: Ag and FVIII in HA blood donors with different genders and blood types. Values are mean ± SD. Non-O-group, n = 24; O-group, n = 17; Female, n = 18; male, n = 23.

The results of vWF: Ag and FVIII:C in HA blood donors with different genders and blood types were reported in Fig 2. The well-known distinctions in vWF: Ag and FVIII:C between non-O and O blood types were also observed in HA blood donors, with significantly higher levels in non-O than in O individuals. In HA blood donors, vWF: Ag was approximately 26% higher in non-O-group than in O-group (1.07±0.21 IU/mL *vs*. 0.79±0.25 IU/mL; p*<*0.001), and FVIII:C was about 9% higher in non-O-group than in O-group (0.68±0.09 IU/mL *vs*. 0.62±0.09 IU/mL; *p* = 0.037). Nevertheless, no significant difference in vWF:Ag and FVIII:C levels was observed between male and female (Fig 2B).

## Discussion

During continuous stay at HA, people may develop several physiological responses to make it possible to live in a low O_2_ environment, such as increasing in RBC (Table 2) and hemoglobin concentration ^[^^12^^–^^13^^]^, rising in platelet aggregation count and fibrinogen levels ^[^^4^^,^ ^14^^]^, and changes in coagulation factors ^[^^5^^,^ ^14^^,^ ^15^^]^. All of these could bring about changes in blood composition. In this study, we investigated the influences of HA on FFP quality and evaluated whether the current quality requirement of FFP is suitable for HA blood donors.

In China, FVIII:C and the content of total plasma proteins are the two most important indicators mentioned in the current quality requirement of FFP. In order to fully understand the influence of HA on the quality of FFP, we evaluated the content of total plasma proteins and the levels of clotting factors in FFP of different altitude blood donors. Total plasma protein and FVIII were robust analyzed by ROC curve (S1 Fig and S1 Table). When compared with LA blood donors, the total protein content of HA blood donor was significantly decreased (65.2±8.9 g/L *vs*. 57.2±6.3 g/L; *p<*0.001), although it still met the current Chinese quality requirement of FFP (≥ 50 g/L). We found that the screening test measurements (PT, aPTT and TT) of coagulation system were significantly higher in HA than in LA blood donors (Table 3). Similar results have been confirmed by Singh and colleagues, which showed that PT and TT significantly prolonged in HA subjects. In addition, Pichler Hefti *et al*. found PT and aPTT revealed a small-magnitude but no significant increase with increasing altitude [5]. By contrast, Kotwal and colleagues showed aPTT reduced in HA volunteers [14].

Our study also showed that most of the coagulation factors (including FII, FV, FVII, FVIII, FIX, FX, FXI and FXII) displayed significant decreases in HA blood donors, whereas Fbg and D-dimers statistically increased (p < 0.05). To the best of our knowledge, we are unaware of any similar published results. Singh *et al*. showed that the levels of FV and FVIII increased with increasing altitude, while FXII decreased [15]. It must be pointed out that the mean FVIII:C in HA blood donors was only 0.64±0.10 IU/mL, which was approximately 8.6% lower than that of current Chinese quality requirement (≥ 0.70 IU/mL). In our study, FVIII:C of 11 LA blood donors (23.9%) were < 0.70 IU/mL, whereas up to 70.7% of the HA blood donors (29 of 41 HA blood donors) didn’t meet the current quality requirement.

It seems to be contradictory to that HA is a risk factor for thrombosis, since we found most of the coagulation factors dramaticly decreased, and PT and aPTT notably prolonged in HA FFP. One possible mechanism for increased thrombosis at HA is hypercoagulability [16–18]. Consistent with other reports, we found red blood cells significantly increased in HA blood. More red blood cells lead to hyperviscosity and lower blood flow velocity. Although the number of platelets decreased, yet aggregation and adhesion of platelets significantly increase at HA. As a consequence, HA residents are in a relatively hypercoagulable state. The increasing TT and D-dimers (Table 3 and Fig 1D) implied the activation of the fibrinolytic system which contributed further to the decrease of coagulation factors since plasminogen digests FV and FVIII. Therefore, the decrease of coagulation factors may be a compensatory mechanism as a result of hypoxia at HA.

Consistent with previous reports [14], we also confirmed that anticoagulant factors (PC, PS and ATIII) showed no significant changes (*p>*0.05).

Furthermore, numerous studies generally reported that plasma vWF and FVIII levels were significantly lower in O-group than non-O-group individuals [19–21].Similarly, our study showed vWF: Ag and FVIII:C were significantly lower in O-group than in non-O-group in HA blood donors (*p<*0.05). In another large-scale study on LA blood donors, we also found the levels of vWF and FVIII were significantly lower in O-group than non-O-group [22]. This could also provide reference for FFP preparation. Otherwise, Conlan et al. [23] reported that FVIII and vWF levels are significantly higher in females than in males, but our study showed there were no gender differences in FVIII and vWF among HA blood donors (Fig 2B).

Naturally, some limitations of the study should be considered. One potential limitation was that the sample sizes of LA and HA blood donors were small (46 LA and 41 HA blood donors) in the current study. It was quite difficult to recruit HA volunteers. Because of the national belief, almost no Tibetans would like to donate blood. However, numerous similar studies were also conducted with a small sample size (less than 100).

In conclusion, this study evaluated the content of total plasma protein and the levels of coagulation and anticoagulation factors in FFP of HA and LA blood donors. Our study clearly suggested that altitude significantly influenced the quality of FFP. The content of total plasma protein was significantly lower in HA than LA blood donors. Except Fbg and D-dimers, all other coagulation factors (i.e. FII, FV, FVII, FVIII, FIX, FX, FXI, FXII, and vWF: Ag) observably decreased with increasing altitude. It should be pointed out that FVIII:C of 29 HA blood donors (70.7%) were less 0.7 IU/ml which didn’t meet the current Chinese quality requirement for FFP. And there were no significant changes in the anticoagulation factors (PC, PS and ATIII) between HA and LA blood donors. In HA blood donors, vWF: Ag and FVIII:C were significant higher in non-O-group than in O-group. Taken together, this study suggested that the altitude could significantly influence the quality of FFP and the current Chinese quality requirement of FFP may not be suitable for HA. Further studies should expand the sample size to validate the findings and set up a special quality requirement for HA FFP, which will help to improve HA blood transfusion efficiency.

## Supporting information

S1 Fig ROC curve for total protein content and FVIII:C. (TIF)

S1 Table ROC curve analysis. (DOC)

## References

[1] Liu Y, Zhang JH, Gao XB, Wu XJ, Yu J, Chen JF, et al Correlation between blood pressure changes and AMS sleeping quality and exercise upon high-altitude exposure in young Chinese men. Mil Med Res. 2014; 1:19 doi: 10.1186/2054-9369-1-19 25722875 10.1186/2054-9369-1-19 PMC4340834

[2] Jha SK, Anand AC, Sharma V, Kumar N, Adya CM. Stroke at high altitude: Indian experience. High Alt Med Biol. 2002;3:21–27 doi: 10.1089/152702902753639513 12006161 10.1089/152702902753639513

[3] Gambhir RPS, Anand V, Khatana SS, Bedi VS. A Brief Review of High Altitude Thrombosis. Ind J Vasc Endovasc Surg. 2014;1(1): 20–23.

[4] Vij AG. Effect of prolonged stay at high altitude on platelet aggregation and fibrinogen levels. Platelets. 2009;20:421–427 doi: 10.1080/09537100903116516 19658003 10.1080/09537100903116516

[5] Pichler Hefti J, Risch L, Hefti U, Scharrer I, Risch G, Merz TM, et al Changes of coagulation parameters during high altitude expedition. Swiss Med Wkly. 2010;140:111–117. doi: smw-12910 19950043 10.4414/smw.2010.12910

[6] Iorio A, Basileo M, Marchesini E, Palazzesi GP, Materazzi M, Marchesi M, et al Audit of the clinical use of fresh-frozen plasma in Umbria: study design and results of the pilot phase. Blood Transfu. 2008;20:211–219 10.2450/2008.0042-07 PMC2626902 19112736

[7] Schofield WN, Rubin GL, Dean MG. Appropriateness of platelet, fresh frozen plasma and cryoprecipitate transfusion in New South Wales public hospitals. Med J Australia. 2003; 178: 117–121 12558482 10.5694/j.1326-5377.2003.tb05101.x

[8] Stanworth SJ, Grant-Casey J, Lowe D, Laffan M, New H, Murphy MF, et al The use of fresh-frozen plasma in England: high levels of inappropriate use in adults and children. Transfusion. 2011; 51:62–70 doi: 10.1111/j.1537-2995.2010.02798.x 20804532 10.1111/j.1537-2995.2010.02798.x

[9] National Health and Family Planning Commission of the People’s Republic of China (2000). The Technical Criterion of Clinical Blood Transfusion. URL http://www.nhfpc.gov.cn/yzygj/s3589/200804/adac19e63a4f49acafab8e0885bf07e1.shtml (Accessed on 18/01/2016)

[10] National Health and Family Planning Commission of the People’s Republic of China (2012). Quality requirements for whole blood and blood components. URL http://www.nhfpc.gov.cn/zwgkzt/s9493/201207/55380/files/a1bb8c98233146408357e804b09013b4.PDF. http://www.moh.gov.cn/zwgkzt/s9493/201207/55380.shtml (Accessed on 12/06/2016)

[11] National Health and Family Planning Commission of the People’s Republic of China (2011). Whole blood and component donor selection requirements. URL http://www.nhfpc.gov.cn/zhuzhan/zcjd/201304/19aefe515bbc4c6d86bb64a389195a38/files/d91b6b677dc341e08b70e44d5176273e.PDF. (Accessed on 03/12/2015)

[12] Frisancho AR. Developmental functional adaptation to high altitude: review. Am J Hum Biol. 2013; 25:151–168. doi: 10.1002/ajhb.22367 24065360 10.1002/ajhb.22367

[13] Weitz CA, Garruto RM. Growth of Han migrants at high altitude in central Asia. Am J Hum Biol. 2004; 16:405–419 doi: 10.1002/ajhb.20042 15214059 10.1002/ajhb.20042

[14] Kotwal J, Apte CV, Kotwal A, Mukherjeed B, Jayaram J. High altitude: a hypercoagulable state: results of a prospective cohort study. Thromb Res. 2007; 120:391–397 doi: 10.1016/j.thromres.2006.09.013 17084442 10.1016/j.thromres.2006.09.013

[15] Singh I, Chohan IS. Blood coagulation changes at high altitude predisposing to pulmonary hypertension. Brit Heart J. 1972; 34:611–617 5040260 10.1136/hrt.34.6.611 PMC458508

[16] Theusinger OM, Baulig W, Asmis LM, Seifert B, Spahn DR. In vitro factor XIII supplementation increases clot firmness in Rotation Thromboelastometry (ROTEM). Thromb Haemostasis. 2010;104:385–391 20431856 10.1160/TH09-12-0858

[17] Kohler HP. Interaction between FXIII and fibrinogen. Blood. 2013;121:1931–1932. doi: 10.1182/blood-2013-01-479055 23493770 10.1182/blood-2013-01-479055

[18] Maher JT, Levine PH, Cymerman A. Human coagulation abnormalities during acute exposure to hypobaric hypoxia. J Appl Physiol. 1976; 41:702–706. 993158 10.1152/jappl.1976.41.5.702

[19] Miller C, Haff E, Platt S, Rawlins P, Drews C, Dilley AB, et al Measurement of von Willebrand factor activity: relative effects of ABO blood type and race. J Thromb Haemost. 2003; 1: 2191–2197. 14521604 10.1046/j.1538-7836.2003.00367.x

[20] Favaloro EJ, Soltani S, Mc Donald J, Grezchnik E, Easton L, Favaloro JW. Reassessment of ABO Blood Group, Sex, and Age on Laboratory Parameters Used to Diagnose von Willebrand Disorder. Am J Clin Pathol. 2005; 124: 910–916. 16416741

[21] Kokame K, Sakata T, Kokubo Y, Miyata T. von Willebrand factor‐to‐ADAMTS13 ratio increases with age in a Japanese population. J Thromb Haemost. 2011; 9: 1426–1428. doi: 10.1111/j.1538-7836.2011.04333.x 21535397 10.1111/j.1538-7836.2011.04333.x

[22] Wang ZK, Dou MM, Du X, Ma L, Sun P, Cao HJ, et al Influences of ABO blood group, age and gender on plasma coagulation factor VIII, fibrinogen, von Willebrand factor and ADAMTS13 levels in a Chinese population. PeerJ. 2017; 5: e3156 doi: 10.7717/peerj.3156 28382235 10.7717/peerj.3156 PMC5376111

[23] Conlan MG, Folsom AR, Finch A, Davis C, Sorlie P, Marcucci G, et al Associations of factor VIII and von Willebrand factor with age, race, sex, and risk factors for atherosclerosis. The Atherosclerosis Risk in Communities (ARIC) Study. Thromb Haemostasis. 1993;70: 380–385. 8259533

