# Supplementary figures and images for: The quality changes in fresh frozen plasma of the blood donors at high altitude
Source: PLoS One. 2017 Apr 21;12(4):e0176390. doi: 10.1371/journal.pone.0176390 (PMC5400266; doi:10.1371/journal.pone.0176390)

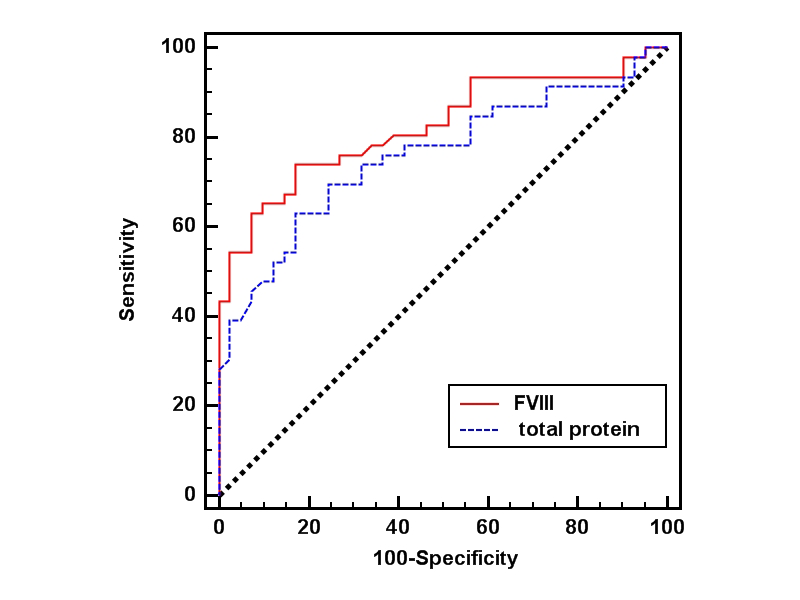

Supplement: S1 Fig — (TIF) [file pone.0176390.s001.tif]
