# Supplementary material for: The quality changes in fresh frozen plasma of the blood donors at high altitude
Source: PLoS One. 2017 Apr 21;12(4):e0176390. doi: 10.1371/journal.pone.0176390 (PMC5400266; doi:10.1371/journal.pone.0176390)
Supplement: S1 Table — (DOC) [file pone.0176390.s002.doc]

**S1 Table.** ROC curve analysis

| Analyte | AUC* | Sensitivity | Specificity | PPV^#^ | p-value |
| --- | --- | --- | --- | --- | --- |
| FVIII | 0.824 | 73.9 | 82.9 | 78.6 | < 0.0001 |
| Total protein | 0.759 | 63.0 | 83.9 | 98.7 | < 0.0001 |

*AUC, Area under the ROC curve; ^#^PPV, positive predictive value.
